# The IGF System and Aging

**DOI:** 10.1210/endrev/bnae029

**Published:** 2024-10-17

**Authors:** Cheryl A Conover, Claus Oxvig

**Affiliations:** Division of Endocrinology, Mayo Clinic, Rochester, MN 55905, USA; Department of Molecular Biology and Genetics, Aarhus University, 8000 Aarhus, Denmark

**Keywords:** insulin-like growth factors, aging, pulmonary fibrosis, Alzheimer's disease, age-related macular degeneration

## Abstract

There is strong evidence that IGF signaling is involved in fundamental aspects of the aging process. However, the extracellular part of the IGF system is complex with various receptors, ligand effectors, high-affinity IGF-binding proteins, proteinases, and endogenous inhibitors that all, along with their biological context, must be considered. The IGF system components are evolutionarily conserved, underscoring the importance of understanding this system in physiology and pathophysiology. This review will briefly describe the different components of the IGF system and then discuss past and current literature regarding IGF and aging, with a focus on cellular senescence, model organisms of aging, centenarian genetics, and 3 age-related diseases—pulmonary fibrosis, Alzheimer disease, and macular degeneration—in appropriate murine models and in humans. Commonalities in mechanism suggest conditions where IGF system components may be disease drivers and potential targets in promoting healthy aging in humans.

## Graphical Abstract

**Graphical Abstract:**
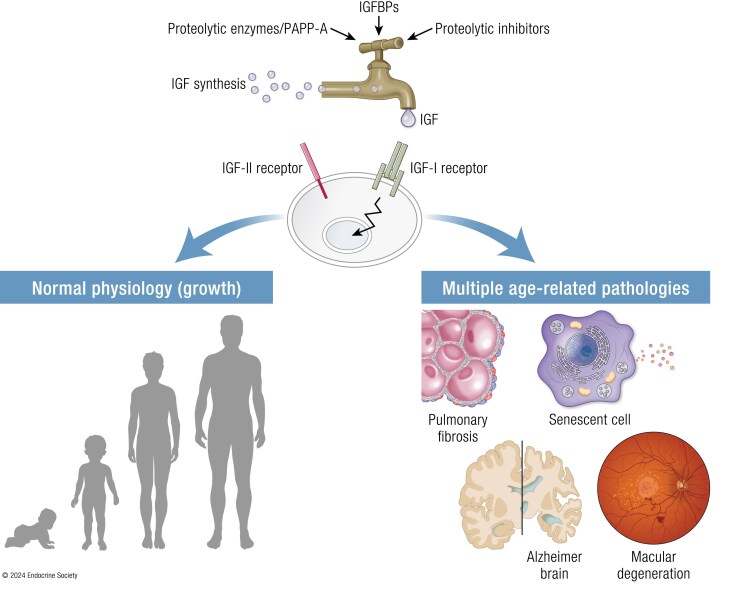

**ESSENTIAL POINTS**

- The IGF system is evolutionarily conserved and involved in fundamental aspects of aging
- This review covers the involvement of IGF system in cellular senescence, model organisms of aging, centenarian genetics, and 3 human age-related diseases—pulmonary fibrosis, Alzheimer disease, and macular degeneration
- IGF system components including receptors, ligands, binding proteins, proteinases, and inhibitors may be disease drivers and potential targets in promoting healthy aging

**Abbreviations**
Aβ: amyloid-β
AD: Alzheimer disease
ALS: acid-labile subunit
AMD: age-related macular degeneration
BBB: blood-brain barrier
CSF: cerebrospinal fluid
IGF-IR: type I IGF receptor
IGF-IIR: type II IGF receptor
IPF: idiopathic pulmonary fibrosis
IRS: insulin-receptor substrate
PAPP: pappalysin metalloproteinase
PI3K/Akt: phosphatidylinositol 3-kinase
SASP: senescence-associated secretory phenotype
STC: stanniocalcin

Experimental data generated during the past decades suggest that IGF signaling is involved in fundamental aspects of the aging process. However, the actual role of intracellular signaling cascades associated with IGF signaling is incompletely understood. Furthermore, molecular mechanisms that regulate IGF activity in the extracellular compartment also remain to be better understood and connected to the aging process.

The IGF system is complex with various receptors and receptor-triggered intracellular signaling pathways, ligand effectors, binding proteins, proteinases, and endogenous inhibitors that all need to be considered, along with the specific context in which they are involved. These components are evolutionarily conserved, underscoring the importance of the IGF system in physiology and pathophysiology. A brief summation of these components is presented in this introductory section, but the reader is referred to excellent general reviews of the IGF system for further details of how they together contribute to regulating IGF activity (1‐6).

##  

### IGF Receptors

The type I IGF receptor (IGF-IR) has two α- and two β-subunits linked by disulfide bonds. The α-subunit is responsible for ligand binding. The β-subunit has tyrosine kinase activity when ligand is bound, which initiates several intracellular phosphorylation cascades on adaptor proteins such as insulin-receptor substrates (IRSs) that activate signaling pathways, principally phosphatidylinositol 3-kinase (PI3K/Akt) and MAPK. IGF-IR exhibits high sequence and structural similarity with the insulin receptor, and binds IGF-I with high affinity, IGF-II with lower affinity, and insulin with very low affinity. The IGF-IR mediates mitogenesis, differentiated functions, and cell survival in various cell types. Because of their structural similarity, 1 α/β half molecule of each of the receptors can form IR/IGF-I-R heterodimeric hybrids, which bind IGF-I with higher affinity than insulin and are more mitogenic than metabolic in their signaling outcomes. The type II IGF receptor (IGF-IIR) also known as the cation-independent mannose-6-phosphate receptor, has a large extracellular domain that can bind various substrates including IGF-II. IGF-IIR principally functions to attenuate IGF signaling by binding and internalizing IGF-II for degradation and transporting lysosomal enzymes to lysosomes, and thus it not directly engaged in signaling.

### IGF Ligands

The IGFs (IGF-I and IGF-II) are peptides structurally related to insulin. They consist of continuous A, B, C, and D domains, unlike the 2-chained insulin molecule, which lacks the C domain. IGF-I is expressed in the liver under GH control, and this organ is the principal contributor to circulating IGF-I. However, IGF-I is also expressed in most tissues for autocrine/paracrine effects. This review will focus on IGF-I and IGF-IR, although IGF-II will be included where relevant.

### IGF Binding Proteins

There are 6 high-affinity IGF binding proteins (IGFBP-1 through IGFBP-6) with ubiquitous presence in the circulation and in tissues. They function to modulate IGF binding to the IGF-IR and thus receptor activation but have no influence on insulin signaling. Although structurally similar to each other with N- and C-terminal domains, both engaged in IGF binding, there are differences that influence context-dependent properties. In principle, IGFBPs bind and inhibit IGF action. However, posttranslational modifications, such as proteolytic cleavage, phosphorylation, and binding to extracellular matrixes and cell membranes that occur mostly in the less structured linker region of the IGFBPs, can individualize their effects on cells and tissues. In the circulation, the liver-derived acid-labile subunit (ALS) allows IGFBP-3 and, to a lesser extent IGFBP-5, bind to IGF to form a ternary complex that increases the half-life of IGF in the circulation.

### IGFBP Proteinases

Proteolytic cleavage of IGFBP-3 was first observed in the serum of pregnant women (7), and, subsequently, matrix metalloproteases, kallikreins, cathepsin D, and complement component C1 were found to cleave IGFBP-2 through IGFBP-6 with varying specificities in vitro. However, so far only the pappalysin metalloproteinases, PAPP-A and PAPP-A2, appear to be physiological regulators of IGF signaling by their abilities to cleave IGFBP-2 (PAPP-A), IGFBP-3 (PAPP-A2), IGFBP-4 (PAPP-A), and IGFBP-5 (PAPP-A and PAPP-A2) at a single site in the IGFBP linker domain.

Although PAPP-A was first identified as 1 of 4 antigens present at high levels in the plasma of pregnant women (8), its enzymatic activity was only later discovered in medium conditioned by human fibroblasts, osteoblasts, vascular smooth muscle cells, and various other normal and cancer cell types. Secreted PAPP-A tethers to the surface of cells by binding proteoglycans and thus can activate IGF by cleaving the inhibitory IGFBPs in the pericellular environment to cause IGF-IR activation. PAPP-A-induced enhancement of local IGF-I action through cleavage of IGFBP-4 has been demonstrated in several in vitro and in vivo systems. The physiological relevance of PAPP-A cleavage of IGFBP-2 and -5 is less clear. The homolog, PAPP-A2, cannot bind cell surfaces and thus appears to be more important in the circulation, whereas PAPP-A is more important in the tissue microenvironment.

The stanniocalcins (STC1 and STC2) were recently discovered to inhibit pappalysin activity potently. STC1 shows high-affinity reversible binding to PAPP-A and PAPP-A2 (9). STC2, on the other hand, binds covalently to PAPP-A and PAPP-A2, and thus inhibits irreversibly (10). Recent structural data obtained by cryoelectron microscopy has revealed the complex inhibitory mechanism between PAPP-A and STC2 (11). The stanniocalcins appear to have additional biological functions separate from pappalysin inhibition, although biochemical details are currently scarce.

## Cell-based Studies Implicating the IGF System in Aging

### Cellular Senescence

Cellular senescence is a complex response of cells to various stressors (12, 13). It is characterized by stable arrest of proliferation, resistance to apoptosis, and expression of a senescence-associated secretory phenotype (SASP). The biological effects of the SASP proteins in the tissue microenvironment can be autocrine, thus reinforcing senescence of the secreting cell. SASP proteins can also affect nearby nonsenescent cells in a paracrine manner, or they can function to attract immune cells (14, 15). For these and other reasons, SASP proteins have been suggested as potential targets for the prevention of several age-related conditions (16‐18).

### IGFs and Cellular Senescence

There is limited specific knowledge about the IGFs in senescence. On one hand, it is widely established in many cells that acute treatment with IGF-I stimulates proliferation. On the other hand, prolonged IGF-I treatment promotes premature cellular senescence (19‐21), and IGF-I has also been reported to enhance cellular senescence in confluent cell culture (22). Although IGF-I itself may not be considered a SASP protein, the SASP response of a particular cell can be modulated by IGF signaling, for example in dermal fibroblasts (23). Several of the IGF regulatory proteins discussed previously have been reported as part of the SASP response of several different cell types. These proteins may function indirectly by modulating IGF bioactivity and hence IGF signaling, they may function independent of IGF signaling, or they may impact the secreting cell or its neighboring cells by both mechanisms.

### IGFBPs and Cellular Senescence

All 6 IGFBPs were identified in a gene set of SASP proteins across tissues (24), and 4 of the IGFBPs were identified in a separate screen of several cell types (25, 26). Most studies, however, report and analyze more narrow subsets of SASP proteins. For example, SASPs from preadipocytes and lung fibroblasts have been shown to contain elevated levels of proteolytically active PAPP-A that can spread senescence to neighboring nonsenescent cells as a soluble component and in association with the outer membrane of extracellular vesicles (27, 28). IGFBP-4 has also been reported in various SASPs, where it would potentially serve as a substrate for PAPP-A and, thus, increase IGF activity (29, 30). Increased IGFBP-3 accumulation in senescence has been proposed to bind and inhibit IGF activity (31), and IGFBP-5 has been shown to induce cell senescence (32, 33). However, the function of IGFBP-5 in cellular senescence is less clear because it could have negative or positive effects on IGF signaling and could have IGF-independent effects as well (34).

Recently, STC1 was identified as a SASP protein from multiple cell types and various means of senescence induction, and it was noted that, although STC1 quantitatively appeared as 1 of the top candidates, it had not previously been reported as a SASP protein (25). This finding was corroborated by data showing that STC1 highly correlates with chronological human age (35). Because STC1 binds with high affinity to PAPP-A, it will obviously negatively affect PAPP-A activity and hence the ability of PAPP-A to cleave IGFBPs and promote IGF signaling—depending on local concentrations, molar ratios, and distribution in the tissue microenvironment. However, other potential activities of STC1 may also be important in this context.

## Model Organisms of Aging and Age-related Diseases

### IGF and Aging in Invertebrates

The IGF signaling pathway is a key determinant of aging. Increased IGF signaling has been associated with aging and many age-related diseases, and disruption of this signaling pathway prolongs lifespan in many different species. Hypomorphic mutations in the round worm (*Caenorhabditis elegans*) decreases the activity of DAF-2, which encodes a receptor similar to the IGF-IR, more than doubles the lifespan (36). Worm mutations affecting downstream AGE-1 (a PI3K ortholog) extends lifespan via inactivation of DAF-16, a FOXO transcription factor (37, 38). In the fruit fly (*Drosophila melanogaster*), inhibition of IGF-IR-like signaling through a mutation in an IRS also significantly increases lifespan (39, 40).

### IGF and Aging in Mice

Being important for optimal growth early in life but detrimental to the organism later in life, IGF signaling fits with the “antagonistic pleiotropy theory of aging” (41). This has been studied in many different mouse strains with direct or indirect effects on IGF signaling.

#### IGF-IR

Homologous disruption of the IGF-IR gene in mice is embryologically lethal (42). However, mice with reduced IGF-I signaling exhibit significantly increased lifespan and healthspan, which appear to vary with genetic background and sex. Longevity for mice heterozygous for IGF-IR inactivation varied, with 5% to 33% increases depending on background and laboratory conditions (43‐45). This extended lifespan was only observed in the female mice. Late-life targeting of the IGF-IR with a monoclonal antibody also improved lifespan and healthspan in female mice (46). The reason for sexual dimorphism in these studies is unclear (47). Lifespan of mice lacking either the IRS-1 or IRS-2, the major intracellular effectors of IGF-IR signaling was also studied. Selman and colleagues showed that IRS-1 null mice were long-lived, whereas IRS-2 null mice were shorter-lived than wild-type mice. Heterozygous IRS-1 and IRS-2 mice showed normal lifespans (48, 49). Taguchi et al (50). reported that mice heterozygous for a null-mutation in IRS-2 showed an increased lifespan; however, these findings could not be replicated by others (51). Different diets and effects on metabolism potentially explain the reason for the different impact of reduced IRS-2 on longevity in these studies (52). Mice with deficiencies in GH (Ames, Snell, GH receptor gene deletion) have low circulating IGF-I and also live longer than wild-type littermates (53). Comparative analysis of hepatic gene expression levels in these long-lived dwarf mice confirmed down-regulation of IGF-I expression (54). However, these mouse models of longevity are multifaceted (55), and it is unclear if they actually have reduced local IGF signaling (56).

#### PAPP-A

PAPP-A acts to enhance local IGF bioavailability in multiple tissues. Thus, in agreement with the antagonistic pleiotropy theory of aging, PAPP-A gene deletion in mice produced a smaller-sized mouse with many beneficial traits, including a remarkable extension of lifespan in both male and female mice by ∼30% with no effect on circulating IGF-I levels (57). A significant increase in lifespan was also seen with PAPP-A knockout mice on a high-fat diet and when PAPP-A gene expression was conditionally knocked out in adult mice of normal size (57‐59). Furthermore, PAPP-A knockout mice showed resistance to atherosclerotic plaque progression, resistance to visceral obesity while on a high-fat diet, delayed thymic involution with persistence of a youthful T-cell phenotype in aged mice, prevention of sarcopenia with age, enhanced insulin sensitivity, and resistance to bleomycin-induced pulmonary fibrosis (4). In atherosclerosis, visceral obesity, and pulmonary fibrosis, the beneficial effects have been replicated with administration a monoclonal antibody, which inhibits PAPP-A activity (60‐62). Interestingly, studies where senescent cells were deleted in mice produced a phenotype very similar to studies where PAPP-A was deleted in mice. Examples of these associations are presented in Table 1. It remains to be determined whether there is a cause/effect relationship.

**Table 1.** PAPP-A and cell senescence

Effects on aging and age-related disorders	Delete senescent cells	Delete PAPP-A
Increase lifespan	Yes (63, 64)	Yes (57-59, 65)
Impede atherosclerosis	Yes (66)	Yes (67‐69)
Prevent visceral obesity	Yes (70, 71)	Yes (72)
Delay thymic involution	Yes (73, 74)	Yes (75)
Prevent sarcopenia	Yes (76)	Yes (77)
Enhance insulin sensitivity	Yes (78)	Yes (79)
Prevent pulmonary fibrosis	Yes (80, 81)	Yes (28, 62)

### Pulmonary Fibrosis in Mice

Pulmonary fibrosis is a component of many interstitial lung diseases, including idiopathic pulmonary fibrosis (IPF). This debilitating condition is the result of exaggerated deposition of extracellular matrix leading to irreversibly distorted lung architecture and respiratory insolvency.

#### IGFs

IGF-I has been implicated in the pathogenesis of pulmonary fibrosis. Thus, IGF-I is increased after bleomycin-induced lung injury in mice associated with increased extracellular matrix deposition and pro-survival signaling through PI3K/Akt (82‐85). Blockade of the IGF-I pathway with an IGF-IR antibody increased apoptosis and decreased fibrosis (80). Hernandez et al (86). documented upregulation of IGF-I via TGF-β in lung fibroblasts and found that increased IGF-I expression correlated with decreased pulmonary function in mice following bleomycin-induced injury. Administration of an IGF-IR inhibitor slowed the progression of murine lung fibrosis.

#### IGFBPs

Dysregulated IGFBPs may be a key factor in the initiation and progression of pulmonary fibrosis. IGFBP-3, IGFBP-4, and IGFBP-5 expression is increased in lungs of patients with IPF, and cultured IPF fibroblasts secrete and deposit IGFBP-3 and IGFBP-5 into the extracellular matrix (83, 84). Although IGFBP-3 and IGFBP-5 are able to bind IGF, it is possible that they also have IGF-independent roles in pulmonary fibrosis. IGFBP-5, in particular, has been implicated in promoting pulmonary fibrosis through increased collagen and fibronectin expression and induction of fibroblast/myoblast transdifferentiation independent of IGF-I (85, 87‐89). IGFBP-5 can also function in an autocrine manner to increase its own expression, further potentiating fibrosis.

#### PAPP-A

PAPP-A is expressed in mouse lung fibroblasts and is induced after intratracheal administration of bleomycin. Inhibition of PAPP-A in vivo through PAPP-A gene deletion or pharmacologically with a monoclonal antibody markedly reduced the progression of fibrosis in a bleomycin-induced lung injury model, as measured by significantly decreased extracellular matrix expression and improved lung histology (62). Local IGF receptor activity was also significantly reduced, indicating indirect reduction of IGF signaling through PAPP-A inhibition.

### Murine Models of Alzheimer's Disease

Alzheimer disease (AD) is a progressive neurodegenerative disease of age with no effective preventative or treatment approaches. Several mouse models have been used to investigate the role of the IGF system in AD (90‐93).

#### Brain IGF-I

Suppression of local IGF signaling in AD mice has been found to reduce plaque formation in the brain and delay neurodegeneration and behavioral changes. Numerous studies indicate that experimental AD in mice responds positively to reduction of local IGF signaling in the brain through genetic means. Thus, deletion or knock-down of IGF-IR or intracellular mediators of IGF signaling in various mouse models of AD resulted in a significant attenuation of pathological β-amyloid, neuro-inflammation, neuro-degeneration, and subsequent behavior changes (94‐98). Neuron-specific knock-out of IGF-IR correlated with decreased neuro-inflammation and improved spatial memory in mouse models of AD (94, 95, 97), and genetically ablating IGF-IR in neurons of the aging brain was protective against the neuro-inflammation, amyloid-β (Aβ) proteotoxicity, and memory impairment induced by intracerebroventricular injection of amyloidogenic Aβ oligomers (97). Furthermore, reduced IGF signaling helped to maintain autophagy in neurons with age and promote the clearance of toxic Aβ oligomers (94, 95, 97). Thus, suppression of local IGF-IR signaling in AD mice can reduce plaque formation in the brain and prevent/delay neurodegeneration and associated behavioral changes. Furthermore, long-term blockade rather than enhanced IGF signaling supports neuronal function and neuroprotection (98).

#### Brain IGF-II

IGF-II is the more abundantly expressed IGF in the central nervous system, where it is synthesized by the choroid plexus and meninges in adult rodent brains, and it may have different roles than IGF-I. Chen et al (99) were the first to report a critical role for IGF-II in memory enhancement and consolidation mediated by IGF-IIR, which is highly enriched in neurons and involved in endosomal trafficking and lysosomal targeting. In that study, hippocampal injection of IGF-II enhanced memory retention, whereas anti-IGF-IIR (but not anti-IGF-IR) antibodies completely abolished the memory enhancing effect. To enhance memory, IGF-II must be present when the brain system is active (100, 101). Yu et al (102) also reported that hippocampal IGF-IIR plays a critical role in memory consolidation.

In mouse models of AD, IGF-II administration ameliorated cognitive impairment and reduced toxic Aβ oligomers in the brain via IGF-IIR. Intracerebroventricular IGF-II administration prevented several pathological processes associated with AD (103). Adeno-associated virus-mediated IGF-II expression in the hippocampus, and to a lesser extent, AAV-IGF-I, reduced brain Aβ levels and amyloid plaque in AD mice (104). IGF-II/IGF-IIR appears to facilitate Aβ clearance, however the mechanism is unclear. The large extracellular domain of IGF-IIR has binding sites for various ligands besides IGF-II that may interact to regulate exocytosis.

#### Systemic IGF

Endocrine IGF-I has been shown to be important for spatial learning and memory in old mice (105), and neurodegenerative disorders including AD have been associated with decreased serum IGF-I concentrations. Elevated circulating levels of IGF-I have been associated with beneficial effects on the aging brain and in AD (106‐108). Systemic infusion of IGF-I in AD mice improved cerebrovascular dysfunction, corrected associated behavioral deficits, and promoted clearance of Aβ from the brain (106). Serum IGF-I can enter the central nervous system by a saturable transport system at the mouse blood-brain barrier (BBB) that is influenced by peripheral IGFBPs (109, 110). In particular, serum IGF-I can enter the brain by crossing the BBB at the choroid plexus via the IGF-IR or the transport receptor, megalin, which in turn regulates brain amyloid levels by enhancing the transfer out of Aβ peptides (111). However, the majority of serum IGF is bound to IGFBP-3 and IGFBP-5 in the ternary complex with ALS, which limits transport between compartments. It is not known whether IGFBP proteinases are involved in increasing IGF available for transport. Long-lived Ames dwarf mice with extremely low circulating IGF-I resulting from primary GH deficiency maintain local brain IGF-I expression and active PI3K/Akt signaling and exhibit normal cognitive function (112).

#### PAPP-A

Deletion of PAPP-A in a mouse model of AD provided protection against pathologic and behavioral changes. Compared to AD mice, AD/PAPP-A KO mice had reduced amyloid plaque number in the cortex and hippocampus, along with reduced plaque size; reduced astrocytosis, indicative of neuroinflammation; reduced IGF-I activity in the brain; and improved cognitive behavior (113).

## Human Studies

See Milman et al (114) and Vitale et al (115) for earlier reviews of IGF and human aging.

### Centenarian Genetics

Exceptional human longevity appears to be a result of genetic factors more than environmental factors and is associated with enrichment in allelic variation in IGF pathway genes (Table 2).

**Table 2.** IGF system regulation of aging in humans

Extracellular	IGFs, IGFBPs, PAPP-A
Transmembrane	IGF-IR*^a^*
Intracellular	IRS*^a^*, PI3K*^a^*, FOXO*^a^*

^*a*^Indicates known variants that influence IGF signaling.

#### IGF-IR

Variants in the IGF-IR gene were associated with longevity in an Italian population (116) and in Jewish female centenarians (117). In the latter group, Ashkenazi Jews with exceptional longevity were found to have overrepresentation of a loss-of-function heterozygous mutation in the IGF-IR gene (118). Polymorphic variations in PI3K, Akt, and IRS-2 genes were also associated with human longevity (116, 119, 120).

#### FOXO

FOXO3A belongs to a subfamily of transcription factors implicated in IGF signaling (121). Genetic variation of the FOXO3A gene was strongly associated with long-lived Japanese Americans, Han Chinese, Ashkenazi Jews, and Danish, German, and Italian centenarians (122‐126).

### Age-related Disorders

Multiple studies document the involvement of aberrant IGF signaling in a variety of human age-related pathologies such as osteoporosis, type II diabetes, immune aging, cancer, atherosclerosis, in addition to pulmonary fibrosis, AD, and age-related macular degeneration (AMD) that are discussed here. For ethical considerations, most of the data on the IGF system and aging in humans come from tissue samples at autopsy and analyses of blood and other bodily fluids.

### Pulmonary Fibrosis

Idiopathic pulmonary fibrosis (IPF) is an age-associated lung disease of unknown etiology characterized by exaggerated deposition of extracellular matrix leading to distorted lung architecture, respiratory failure, and death. There are no truly effective treatment options for IPF, thus highlighting the importance of exploring new pathogenic mechanisms that underlie the development of fibrosis and of identifying new therapeutic targets. Senescent cells accumulate in IPF lung tissue, and this accumulation correlated with severity of the disease (127‐130). There was also a highly significant correlation between expression of PAPP-A and disease severity in these tissues (28).

### Alzheimer's Disease

To date, the role of the IGF system in healthy aging and age-related cognitive decline is controversial (131). Lack of consistency in the type of cognitive testing, substantial heterogeneity between cohorts, complicated staging of AD, and, in some cases, lack of well-defined antibodies contributed to the mixed results.

#### Tissue

Postmortem brain tissue indicated insulin and IGF-I resistance in AD (132‐134), with defects in insulin and IGF-I signaling via decreased activation of IRS-1/2. IGF-IIR is localized in neurons of the frontal cortex, hippocampus, and cerebellum in control brains. In AD, labeling of neurons for IGF-IIR was less intense (135) and was associated with altered function of the endosomal-lysosomal system. The reduced IGF-IIR expression is probably from neuronal loss in AD. Using redescription mining, PAPP-A was highly associated with cognitive impairment in AD (136). In human AD brain tissue, senile amyloid plaques intensely immunostain for PAPP-A (113).

#### Blood/cerebral spinal fluid

Although some studies have shown significant association of high circulating IGF-I and better motor and cognitive function in humans, others have not. Meta-analysis of circulating IGF-I failed to detect differences between AD patients and controls (137). Lower circulating IGF-I was associated with better cognitive function in women with exceptional longevity (138). However, circulating concentrations of IGF-I do not necessarily reflect activity of IGF-IR signaling. Indeed, blood or cerebrospinal fluid (CSF) IGF-I levels may not be indicative of local action, especially in aged and AD brain where there is IGF-I resistance (133). Serum proteomic signatures of extreme old age indicated significantly higher expression of IGFBP-2 in centenarians, whereas ALS, which stabilizes IGF-I, was higher in offspring and controls (139). Serum IGF-II levels in AD patients were highly variable across studies. However, serum IGF-II can cross the BBB (140), which is important in humans where, unlike mice, high levels of IGF-II in the circulation are retained in adult life. IGF-II is abundant in CSF (141). However, local expression of IGF-II was decreased in human AD brain (142). Plasma PAPP-A was associated with depressive symptoms in older adults (143).

IGFBP-2 is increased in CSF and plasma of patients with AD (144‐147) and is associated with AD risk (148). In a longitudinal study of a relatively large sample size, McGrath et al related elevated circulating IGFBP-2 to subsequent risk for developing AD (149). This was in a population confirmed to be dementia-free at baseline. Araujo et al (150). found IGFBP-2 to be part of a novel machine learning-based panel of plasma proteins that could predict risk of patients with mild cognitive impairment converting to dementia resulting from AD. IGFBP-2 has been shown to have IGF, integrin, nuclear localization, and heparin-binding domains (151), complicating mechanistic understanding of its function(s) in AD.

### Age-related Macular Degeneration

The macula is a small area in the retina responsible for central vision and perception of fine details. AMD is the main reason for vision loss in the elderly and is the most common cause of irreversible blindness in an aged population. Clinically, there is “wet” neovascular AMD (abnormal growth of blood vessels in the retina) and “dry” AMD (atrophic), the latter of which is associated with the formation of white or yellowish deposits of protein and lipid called drusen. Rodents do not have true maculae, so we need to rely on human data for an understanding of the involvement of the IGF system in AMD pathogenesis.

Local, but not circulating, IGFs can exert multiple effects on retinal cell physiology and pathophysiology such as regulating the inflammatory response (152‐158). Blasiak et al suggested that perturbations in the aging stress response leads to AMD, and that IGF-I is 1 of the critical genes in this response (153). A single nucleotide polymorphism in the IGF-IR gene was found to be significantly associated with increased risk of advanced AMD (159).

#### Tissue

Lambooij et al (160) localized IGF-I and IGF-IR throughout neuroretinal layers in normal human eyes and in AMD. IGF-I is produced by human retinal pigment epithelial cells and activates IGF-IR signaling in photoreceptor cells. IGF-I is neuroprotective during normal physiology (152). However, in neovascular AMD there is overexpression of both IGF-I and IGF-IR in human retinal pigment epithelial cells (155).

#### Blood/ocular fluids

Blood levels of IGF-I were found to be higher in intermediate and advanced AMD than in early AMD or healthy eyes (156). IGFBP-2 and IGFBP-3 predominate in vitreous humor from AMD patients (157). Aqueous humor from neovascular AMD patients had significant increases in IGFBP-2 and IGFBP-6. There was also increased IGF-I (158). The retina is an extension of the central nervous system, and there is increased IGFBP-2 in CSF of AD patients (144, 145). IGFBP-6 has preferential affinity for IGF-II (2), suggesting a possible role for IGF-II in AMD.

## Concluding Remarks

This review of the literature presents compelling evidence of a key role for the evolutionarily conserved IGF system in aging and age-related diseases, and commonalities in mechanism suggest conditions where IGF system components may be potential targets in promoting healthy aging in humans. But there are still multiple gaps in knowledge that will be important to fill before this can be realized. We hope the review will inspire and guide further work on the involvement of the IGF system and its regulators in aging.
